# Vaping in Pregnancy: A Systematic Review

**DOI:** 10.1093/ntr/ntab017

**Published:** 2021-02-04

**Authors:** Robert Calder, Eleanor Gant, Linda Bauld, Ann McNeill, Debbie Robson, Leonie S Brose

**Affiliations:** 1 National Addiction Centre, Institute of Psychiatry, Psychology and Neuroscience (IoPPN), King’s College London, London, UK; 2 Usher Institute, University of Edinburgh, Edinburgh, UK

## Abstract

**Introduction:**

Smoking in pregnancy increases the risk of negative health outcomes. Vaping can be effective for smoking cessation in nonpregnant populations. We conducted a systematic review of vaping in pregnancy, covering prevalence, patterns of use, reasons for use, smoking cessation, and health effects.

**Methods:**

Five academic databases were searched on 17 February 2020. Studies reporting prevalence, patterns, reasons, cessation, or health effects of vaping in pregnancy were included; animal and in vitro studies were excluded. A narrative review was used, with risk of bias assessed using Hoy and colleague’s tool, the Newcastle–Ottawa scale, and the Consolidated Criteria for reporting Qualitative Research.

**Results:**

Twenty-three studies were identified: 11 survey, 7 qualitative, 3 cohort, and 2 secondary analyses of randomized clinical trials. Prevalence of vaping in pregnancy (four studies) was between 1.2% and 7.0% overall, and <1% among nonsmokers. Twelve studies reported patterns of use, but findings were inconsistent. Twelve of 14 studies asking why pregnant women vaped reported that most vaped to reduce or quit smoking. Mixed findings were reported from six studies on smoking cessation. Of three studies with health-related outcomes, two were underpowered and one reported similar birthweights for babies born to nonsmokers and women who vaped, with both higher (*p* < .0001) than the birthweight of babies born to smokers.

**Conclusions:**

There were insufficient data to draw conclusions about prevalence, patterns, and effects of vaping in pregnancy on smoking cessation. The limited literature suggests that vaping in pregnancy has little or no effect on birthweight.

**Implications:**

Smoking causes many negative health outcomes for pregnant women and to babies born to people who smoke. There remains a paucity of research on the effects of vaping in pregnancy. There is, however, the potential for vaping products to reduce the negative health outcomes associated with smoking. More research is needed to develop an evidence base in this area.

## Introduction

Worldwide, tobacco kills over 8 million people per year and is a leading cause of death and disease.^1^ Tobacco is also harmful for pregnant women, and smoking when pregnant increases the risk of adverse outcomes such as low birth weight, miscarriage, neonatal and sudden infant death, perinatal morbidity and mortality, premature delivery, and stillbirth.^2^ There are also associations between smoking in pregnancy and infant behavioral outcomes.^3,4^ Exposure to secondhand smoke (from the expectant father for example) is also associated with lower birth weight.^5^ Many adverse health effects, including socioeconomic inequalities such as higher rates of infant deaths and stillbirths in more deprived groups, could be reduced by lower levels of smoking in pregnancy.^7^

Although many smokers quit when pregnant,^8^ it has been challenging to find effective interventions to support those who find it difficult to stop. A Cochrane review concluded that counseling, financial incentives, and providing feedback improved cessation rates, but that outcomes were affected by the characteristics and context of interventions.^6^ The same review highlighted the lack of effective interventions to help prevent postpartum relapses to smoking.

The evidence of efficacy for pharmacological support for smoking cessation in pregnancy is limited. There is even less evidence in relation to vaping products. Nicotine replacement therapy (NRT) has shown little efficacy in controlled trials^9^; however, trials have mostly used a single NRT product and none have used a combination of a patch with a faster-acting type of NRT, which has been shown to be more effective in pregnant smokers in clinical practice.^10^ Efficacy of NRT in pregnancy is further limited by low adherence^9^ and increased metabolism of nicotine during pregnancy.^11^ There is limited evidence on harms of consuming nicotine, separate from smoking, in pregnancy. The US National Academies of Sciences (NASEM) summarized several animal studies that reported adverse effects from in-utero nicotine delivery on lung development and postnatal lung function and behavior. However, none of the studies assessed dose–response relationships, and the animal models used may not replicate human exposure.^12^

Vaping products have been commercially available since the early 2010s.^13,14^ They typically heat a mixture of propylene glycol and vegetable glycerin, nicotine, and flavorings to deliver nicotine to a person via inhalation of the resulting aerosol.^15^ Vaping products have been increasingly used by people to help them quit smoking. In England, for example, vaping products have been the most commonly used smoking cessation aid in the general population since 2013 and more commonly used than nicotine replacement therapies (NRT) prescribed by a doctor or bought over-the-counter from a shop, varenicline, or behavioral support.^16^ A recent Cochrane review (that did not focus on pregnant smokers) found that there was moderate certainty that vaping products containing nicotine improved quit rates compared with non-nicotine vaping products and with NRT.^17^ There is an increasing scientific consensus that, although not risk free, vaping is substantially less harmful than smoking.^12,16,18^

Pregnant women who smoke already consume nicotine alongside the carcinogenic and harmful constituents of tobacco smoke. Pregnant women who switch to NRT or vaping can reduce their exposure to carcinogens.^19^ UK guidance states that nicotine in the form of NRT can be prescribed during pregnancy.^20^ There is currently no guidance on the potential for vaping products to reduce exposure to harms from smoking for pregnant women.

Vaping products have the potential to be used as a form of pharmacological support for people who are pregnant and who wish to quit smoking, they also have the potential to be used as a reduced harm form of nicotine consumption for those who cannot quit smoking. A Cochrane review of pharmacological interventions for smoking cessation in pregnancy concluded that there was insufficient evidence to determine whether NRT had a positive or negative impact on rates of miscarriage, stillbirth, preterm birth, low birthweight, admissions of babies to neonatal intensive care, or neonatal deaths or whether this affected mean birthweights among infants.^9^ However, in one trial in which children of women who smoked but who had sought cessation help and were followed until 2 years of age reported that those born to women who had been randomized to NRT (compared with placebo) were more likely to have healthy development^21^ possibly due to reduced smoking. To date, there are no comparable reviews of vaping in pregnancy,

A report by NASEM (2018) found no evidence whether or not vaping affected pregnancy outcomes and insufficient evidence on whether maternal vaping affected fetal development.^12,22^

A systematic review on the reproductive outcomes from vaping in pregnancy published in 2019^22^ found no studies on this subject, commenting that animal studies indicated a potential for nicotine from vaping to induce birth defects and to alter birth weight. They also noted that most studies of pregnant women who vaped were confounded by participants who also smoked.

### Objective

The present review sought to systematically review evidence on vaping in pregnancy, to answer five review questions:

1. What is the prevalence of vaping during pregnancy and postpartum?
2. Among people who vape during pregnancy, what patterns of use are identified?
3. Among people who vape during pregnancy, what reasons for use are identified?
4. What are the effects of vaping on smoking cessation or reduction during pregnancy and postpartum?
5. Which health outcomes have been reported in studies of vaping in pregnancy and what findings have been reported for these outcomes?

## Methods

The systematic review protocol was preregistered with PROSPERO on 3 June 2019 and can be found here https://www.crd.york.ac.uk/prospero/display_record.php?RecordID=136150.

### Search Methods

The Cumulative Index to Nursing and Allied Health Literature (CINAHL), Embase, Medline, PubMed, and Maternity and Infant Care Databases (MIDIRS) were searched for literature on vaping in pregnancy with no start date up to 17 February 2020. Full search terms are available in the Appendix.

### Selection of Articles

Articles were included where they were peer-reviewed and reported data on prevalence, patterns of use, reasons for use, cessation effects, or health effects of vaping in pregnancy. Articles that reported data from animal studies, in vitro studies, or studies published in a language other than English, French, German, or Italian were excluded. Titles and abstracts were screened by one author with a subsection screened by a second author. Interrater agreement between authors was measured using Cohen’s kappa.^23^ Two authors completed full-text screening with differences discussed and resolved with a third author.

### Data Extraction and Analysis

Data were extracted from the included studies using the data extraction protocol preregistered with the PROSPERO database of prospectively registered systematic reviews. Titles and abstracts were screened by one reviewer with a second author analyzing a subsection for accuracy. Full-text screening was completed by two reviewers with discrepancies resolved by a third author. Data extraction was completed by one reviewer that covered the following areas: study details, prevalence, smoking behavior (including cessation and reduction), pregnancy outcomes, and barriers and facilitators to vaping.

Data were synthesized in a narrative review. To assess prevalence, we only reported data from studies that were representative of a country or a state. For patterns of use, reasons for use, and effects on smoking cessation, we used both qualitative and quantitative data and reported those types of data separately. To assess health outcomes, we reported quantitative data only.

Included studies were assessed for bias or quality. Hoy and colleagues’ method was used to assess the risk of bias in prevalence studies,^24^ the Newcastle–Ottawa Scale was used for cohort studies,^25^ and the Consolidated Criteria for reporting Qualitative Research (COREQ)^26^ to assess the quality of qualitative studies. Ratings for each study are included in Results for each outcome.

## Results

**Figure 1.**
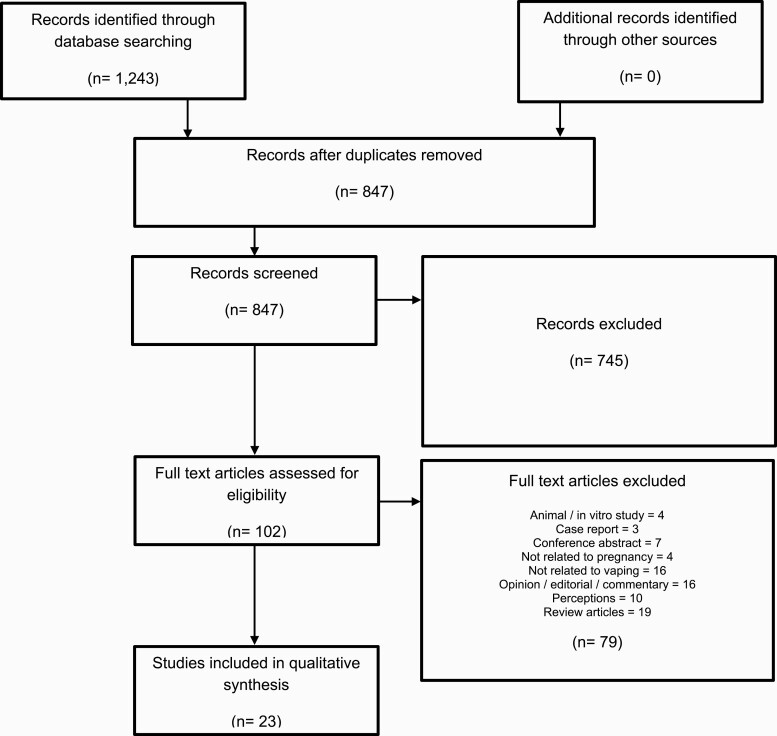
PRISMA flow diagram.

The database search identified 1243 articles, of which 23 were included in the final analysis (Figure 1, Supplementary Table 1). The final Cohen’s kappa coefficient for screening was 0.66, indicating “moderate” agreement.^23^

### Description of Included Studies

#### Location

Seventeen studies were from the United States,^27–43^ three were from the United Kingdom,^44–46^ one of which was based on an analysis of UK online forums,^45^ so that it could have contained information from participants outside of the United Kingdom. One study was from Ireland.^47^ Two studies analyzed online forums are not restricted to a specific country.^48,49^

#### Design

Eight studies reported cross-sectional survey data,^27,28,34,36–38,41,46^ and two studies reported longitudinal survey data.^35,42^ Six studies reporting survey data used samples that were representative of the US population or individual US states.^34–37,41,42^ Additionally, one article reported quantitative data from cross-sectional interviews.^40^

One prospective cohort study collected data from pregnant women who vaped and compared birth outcomes with pregnant women who smoked, who used vaping products and cigarettes (“dual use”), or who used neither vaping products nor cigarettes.^47^ Two articles reported data from a single cohort study of women and babies during and after pregnancy.^29,31^

Two studies used data from randomized controlled trials, one was a secondary analysis of baseline data from a trial of NRT for smoking cessation in pregnancy,^39^ and the other was a secondary analysis of data from a trial of a text message smoking cessation intervention in pregnancy.^30^

Four studies reported qualitative interview or focus group data,^32,33,43,44^ and a further three studies conducted qualitative content analyses of online forums.^45,48,49^

#### Participants

Eight studies recruited women who were pregnant, who were recently pregnant or planning a pregnancy, and who currently or recently smoked.^27,30,32,33,39,43,44,46^ Five studies included women who were pregnant or had recently been pregnant regardless of smoking status.^28,34,36,38,43^ Four used data from representative US surveys that collected information on smoking, vaping, and pregnancy.^35,37,41,42^ One study purposively recruited equal numbers of pregnant smokers and nonsmokers.^40^ Two studies reported data from a single cohort study of pregnant women that tried to recruit equal numbers of smokers, vapers, and nonsmokers.^29,31^ One study recruited pregnant women who vaped and compared them with groups of pregnant women who smoked and who did not smoke.^47^

One study analyzed data from smoking cessation services on staff and on pregnant women who accessed those services.^46^ The three studies of online forum posts analyzed publicly available online discussions about pregnancy but did not confirm pregnancy or smoking status of people posting on those websites.^45,48,49^

### Prevalence of Vaping During Pregnancy and Postpartum

Four studies with low,^37,41^ and moderate,^34,36^ risk of bias, all from the United States, were included in this analysis^34,36,37,41^ (Table 1). Vaping was defined as any use by Kapaya and colleagues and Hawkins and colleagues^34,41^ and as vaping some days or every day by Kurti and colleagues and Liu and colleagues.^36,37^

**Table 1.** Prevalence of Vaping During Pregnancy and Postpartum

Paper ID	Data source	Vaping prevalence	Risk of bias^a^ (Hoy)
Population surveys (all based in the United States)			
Kapaya et al.^34^	Pregnancy Risk Assessment Monitoring System (PRAMS)	Vaping prevalence in peripartum period, defined as any use of vaping products in the 3 mo before pregnancy to 6 mo after delivery: Overall: 7.0%; among those who smoked in past 2 y: 25.1%; among nonsmokers: 2.9%. Broken down by timing: Vaping prevalence in 3 mo before pregnancy: Overall: 10.4%; among those who smoked in past 2 y: 29.8%, among nonsmokers: 6.0%. Vaping prevalence during last pregnancy trimester: Overall: 1.4%; among those who smoked in past 2 y: 5.1%; among nonsmokers: 0.5%. 2–6 mo after delivery: 2.1%; among those who smoked in past 2 y: 8.6%; among nonsmokers: 0.7%.	Moderate risk (4/10)
Hawkins et al.^41^	Pregnancy Risk Assessment Monitoring System (PRAMS)	Any use of vaping products during the last 3 mo of pregnancy was estimated at 1.2% (ranging from 0.6% in New York City to 4.4% in West Virginia) with 0.5% of participants reporting exclusive use of vaping products (with no concurrent smoking). 0.5% of participants who did not smoke at all during pregnancy used vaping products, and 9.7% of people who used cigarettes during their pregnancy also used vaping products.	Low risk (3/10)
Kurti et al.^36^	National survey, Population Assessment of Tobacco and Health (PATH)	Current vaping in pregnancy: Overall: 4.9%; among current smokers: 28.5%; among former smokers: 2.3%; among never smokers: 0. Former vaping in pregnancy: Overall: 18.4%; among current smokers: 44.5; among former smokers: 28.0%; among never smokers: 0.6%. Current use defined as those who vaped fairly regularly and used some days or every day (current established vapers). Or who used some days but not fairly regularly (current experimental vapers).	Moderate risk (5/10)
Liu et al.^37^	National survey, National Health Interview Survey (NHIS)	Current vaping in pregnancy (vaping every day or some days): Overall: 3.6%; among current smokers: 38.9%; among former smokers: 1.3%; among never smokers: 0.3%. Vaping prevalence among nonpregnant women: 3.3% overall, 13.5% among current smokers, 8.8% among former smokers, 0.7% among never smokers.	Low risk (3/10)

^a^Assessment of bias completed using Hoy.^24^

Prevalence of vaping among pregnant women (including smokers and nonsmokers) in the last 3 months of pregnancy was between 1.2%^41^ and 1.4%.^34^ Vaping at any time in pregnancy was between 3.6%^37^ and 7.0%.^34^ Among pregnant smokers prevalence of vaping in the last 3 months of pregnancy was between 5.1%^34^ and 9.7%.^41^ Vaping at any time during pregnancy among smokers was between 25.1%^34^ and 38.9%^37^ (Table 1).

Hawkins and colleagues reported that vaping in the last 3 months of pregnancy ranged from 0.6% in New York City to 4.4% in West Virginia; among pregnant women who did not smoke during pregnancy, 0.5% had used vaping products.^41^ In one large survey,^37^ vaping prevalence among pregnant (3.6%) and nonpregnant (3.3%) women was similar (*p* = .92), whereas prevalence of smoking was significantly lower among pregnant women (8.0%) than nonpregnant women (14.3%, *p* = .01).

All surveys included explanations that questions referred to nicotine vaping, reducing the risk of respondents reporting vaping of other substances.

### Patterns of Use

Ten qualitative studies of poor,^27,38,46^ fair,^29,31^ and good^30,34,36,39,41^ quality reported patterns of vaping among pregnant women. There were two further qualitative studies reporting patterns of vaping that scored 27/32^44^ and 20/32^40^ on the COREQ checklist (Supplementary Table 2).

Vaping frequency was inconsistently reported, and the quality of studies varied (Supplementary Table 2). Among all pregnant women in clinic samples, daily use was reported at 0.6% in one study^38^ and at 3% in another.^27^ A large cross-sectional US survey reported that 66.5% of women who vaped in the last trimester of pregnancy did so once a day or more^41^ and 10.4% used vaping products on 2–6 days per week.^41^ In a cohort study, 20.8% of participants vaped on 10 or more days per month, 29.2% vaped between three and nine times per month, and 50% vaped once or twice per month.^29^ One qualitative study reported that, of 29 women attending smoking cessation services, one had vaped every day in the last 30 days, and three said they vaped occasionally.^46^

Only one study reported on the nicotine content of participants’ vaping products; in this large survey, 38% of pregnant vapers (*n* = 3277) used nicotine and 35% used nicotine-free vaping products^34^ (Supplementary Table 2).

One study that included 16 people who vaped when pregnant was the only study to report flavors used.^40^ Among that group, fruit was the most commonly used flavor followed by candy and mint.

One qualitative study of 30 pregnant and postpartum women reported that they preferred small, discreet vaping products and that they avoided vaping in front of children.^44^ A study of 14 pregnant women who vaped reported that half used prefilled cartridges.^39^ No other study reported type of device used (Supplementary Table 2).

### Reasons for Use

Seven qualitative studies^32,33,43–45,48,49^ and seven survey studies of poor,^27,28,38,46^ and good^30,34,39^ quality reported reasons for vaping among pregnant women. Of the qualitative studies, three studies analyzing data from online forums scored between 12 and 16 of 32 on the COREQ checklist,^45,48,49^ with the other four studies ranging between 23 and 31 of 32^32,33,43,44^ (Supplementary Table 2). The quantitative studies were assessed using the Newcastle–Ottawa scale; four were poor quality and three were good quality (Supplementary Table 2).

Common reasons for vaping were to stop smoking or to prevent a return to smoking^27,28,30,32,34,38,39,43–46,48^ and to reduce harm to themselves, their baby and others.^27,30,32–34,38,39,43,44,46,48^ Other reasons for vaping included being able to vape in smoke-free areas, curiosity, relative price to cigarettes, similar hand-to-mouth action as cigarettes, and taste. One study reported that some participants had quit smoking when pregnant intending to resume smoking postpartum and that vaping had prevented their return to smoking.^45^

An analysis of online forum posts reported that, when quitting smoking, some pregnant women had vaped to avoid nicotine withdrawal which they thought might harm their unborn baby.^49^

### Effects of Vaping on Smoking Cessation

Five quantitative studies of poor,^38^ fair,^42^ and good^30,35,39^ quality and one qualitative study that scored 16 of 32 on the COREQ checklist^45^ were included for this outcome (Supplementary Table 2).

One good-quality longitudinal study of 428 pregnant women who smoked reported that at baseline, 36 women had vaped in the past 7 days and 392 women had not.^30^ At 1-month follow-up, those who vaped had similar odds as those who did not vape, of having quit smoking for 7 d and of having attempted to quit smoking (Supplementary Table 2).

Four studies^35,38,39,42^ reported information on smoking cessation but did not assess associations between vaping and changes in smoking behavior.

Motivation to quit smoking was higher among ever vapers compared with never vapers in one survey^38^ but was similar for current vapers and nonvapers in another^39^ (Supplementary Table 2). The same survey reported that current vapers made more attempts to quit smoking than nonvapers^39^; however, the survey by Mark and colleagues^38^ found that ever vapers had not made more quit attempts than nonvapers.

In a longitudinal survey of women who became pregnant between survey waves, 81% of women who vaped before becoming pregnant quit vaping and 53% of women who smoked before becoming pregnant quit smoking.^35^ The study did not test whether vaping was associated with smoking cessation or reduction (Supplementary Table 2). The same survey also found that 4.5% of participants became pregnant between waves and that this group constituted 2.8% of those who continued to smoke at wave 2, 1.3% of those who moved from smoking to vaping, and 14.5% of those who quit smoking and did not use vaping products suggesting that participants who became pregnant were less likely than those who did not become pregnant to continue smoking or to move to vaping, but more likely to quit smoking without using vaping products.^42^

One study analyzing the content of online forums reported posts from women saying that they had quit smoking when pregnant using vaping products and had then continued to vape to remain abstinent from cigarettes postpartum.^45^

### Health Outcomes

Three articles of fair^29,31^ and good^47^ quality published data on pregnancy and birth health outcomes^29,31,47^ (Supplementary Table 3). Current vaping was defined by Cardenas and colleagues and Clemens and colleagues as people who had vaped within the previous month. McDonnell and colleagues defined current vaping as those who self-reported “current” vaping.

A study from a maternity hospital in Ireland^47^ compared the birthweight, birth centile, gestation and breastfeeding of babies born to women who self-reported current vaping, who smoked (defined as at least one cigarette per day) and vaped, who smoked, and who neither vaped nor smoked during the last trimester of their pregnancy.

The study was well powered and found that the mean birthweight of babies born to mothers who vaped (*n* = 218) in the last trimester of their pregnancy was very similar (within 1 g) to those born to nonsmoking mothers who neither smoked or vaped (*n* = 108; 3470 ± 555 g and 3471 ± 504 g respectively; *p* = .97) and was significantly higher than that birthweight of babies born to mothers who smoked (*n* = 99; 3166 ± 504 g; *p* < .001) (Supplementary Table 3). Similarly, the birth centile of babies born to mothers who vaped and to mothers who did not smoke or vape were similar, and both were significantly higher than those born to mothers who smoked. At discharge, 27.2% of babies born to mothers who smoked were breastfed compared with 48.6% of babies born to mothers who vaped (*p* < .001) and 61.1% for babies born to mothers who neither smoked nor vaped.^47^ For mothers who both vaped and smoked (dual users), the outcomes for birthweight, birth centile, and breastfeeding rates at discharge were similar to those for smokers.^47^

Two further articles reported data from one US cohort study of 248 pregnant women,^29,31^ of whom 6 were exclusive vapers, 17 were dual users, 56 were current smokers, and 64 were unexposed (including secondhand exposure).^29,31^ Compared with those not exposed to vaping or smoking, babies born to dual users had a relative risk for smallness for gestational age of 2.5 (95% confidence interval [CI]: 0.7–8.8), similar to the relative risk of those born to smokers (2.6, 95% CI: 0.9–7.2, Supplementary Table 3). The relative risk among people who had vaped compared with those not exposed was 5.1 (95% CI: 1.2–22.2). However, Cardenas and colleagues (2019) commented on the small sample size, noting that “a well-powered study to detect a twofold to threefold increase in risk of smallness for gestational age, assuming a 12% risk of smallness for gestational age among pregnant women not vaping or smoking (ie, the referent group), would require about 300 participants per group (eg, vaping and cigarette dual users, vapers who don’t smoke, cigarette smokers who don’t vape, and the referent group)” (p. 10). In a subsample of the same study, the presence of biomarkers in hair was analyzed. Raw levels of cotinine and the tobacco-specific nitrosamines NNK and NNAL were higher among dual users than among those not exposed to smoking or vaping, but these differences were nonsignificant, possibly due to small sample sizes.^31^ When splitting the sample by nicotine level, those with higher nicotine levels (which we presume were smokers and dual users) had an increased risk of babies that were small for gestational age (Supplementary Table 3). No comparisons are available for exclusive vapers.

## Discussion

The prevalence of vaping among pregnant women in the studies reviewed here (all of which were based in the United States) was between 1.2% and 7.0%. Vaping was rare among nonsmokers. There was little data on flavors and types of products used or frequency of vaping. There were some interesting findings where vaping had seemingly prevented a return to smoking postpartum, although this was from a qualitative study of online forums, so caution must be used when extrapolating from this finding. As in other populations, pregnant women who vaped were likely to do so to help them stop smoking. There were insufficient data available to assess the efficacy of vaping for smoking cessation in pregnant women. Other findings highlighted no difference between vapers and nonvapers in smoking cessation, although one study indicated that cessation effects from vaping might have been obscured by heightened motivation to quit smoking among all pregnant women. The small literature on birth outcomes of vaping in pregnancy suggest that birthweight and birth centile outcomes from mothers who vape during pregnancy may be similar to those for babies born to mothers who neither smoke or vape, and better than for babies born to mothers who smoke.

Vaping prevalence was similar to estimates of vaping prevalence in the US population of 3.2%,^50^ although estimates and definitions of current vaping vary across studies. The lack of nationally representative data on vaping in pregnancy outside of the United States needs to be addressed, and more research is needed on associations between vaping in pregnancy and smoking cessation and health outcomes. As common reasons for vaping in pregnancy are to stop smoking, prevent a return to smoking, and reduce harms, the apparent uncertainty around the harms or risks of vaping in pregnancy is likely to deter use. Additional studies on the effect of vaping on smoking in pregnancy are urgently needed. Similarly, more well-designed studies measuring birth weight and other maternal and fetal health outcomes are needed to improve the evidence base in this area.

The studies identified here reported mixed findings for smoking cessation, with some suggesting that vaping may help some pregnant women quit smoking and others reporting no differences between smokers who vaped and those who did not. One study found that the health outcomes from vaping in pregnancy may be less severe than those from smoking. There are few studies of vaping in pregnancy, and insufficient data to conduct meta-analyses or to draw reliable conclusions. This can, in part, be attributed to the difficulty of studying new interventions in pregnant populations; and in part due to the relatively new availability of vaping products. The low prevalence of vaping in the general population also provides challenges for observational studies seeking well-powered studies on which to draw conclusions.

Limitations of the review include that it did not search gray literature, that the Cohen’s Kappa level of agreement was moderate, that only one reviewer was used for the majority of the abstract screening, that it did not include animal studies and that studies in languages other than English, French, German, or Italian were included. Also, because of the paucity of literature in this area, it was only possible to present a narrative review and not a meta-analysis of the data.

## Conclusions

The data reviewed here are insufficient to draw any firm conclusions for practice or policy. It appears that vaping has less of a detrimental effect on birthweight outcomes than smoking, so pregnant smokers struggling with smoking cessation could benefit from using vaping products in attempts to quit smoking. However, more research would increase the confidence of this recommendation.

## Supplementary Material

A Contributorship Form detailing each author’s specific involvement with this content, as well as any supplementary data, are available online at https://academic.oup.com/ntr.

ntab017_suppl_Supplementary_Materials

ntab017_suppl_Supplementary_Taxonomy_Form

## Appendix—Search Terms

((exp electronic cigarette/) OR (e-cig*) OR (electronic cig*) OR (ENDS AND Nicotine) OR (electronic nicotine delivery system*) OR ((Nicotine) AND (Vaping* OR Vape* OR Vaporiz* OR Vaporis* OR Vapouris*))) AND((exp pregnancy/ OR exp pregnancy complications/ OR exp maternal health services/ OR exp fetus/ OR exp fetal therapies/ OR exp fetal monitoring/ OR exp perinatal care/ OR exp labor pain OR exp analegsia, obstetrical/ OR exp obstetric surgical procedures/ OR exp infant, newborn/ OR exp postpartum period/ OR exp breast feeding/ OR exp prenatal diagnosis/ OR exp obstetrics/ OR exp prenatal education/)) OR ((breast-feeding education OR parturition OR ante natal OR antenatal* OR pre natal* OR prenatal* OR puerper* OR postnatal* OR postpartum OR post partum OR post natal* OR peripartum OR peri partum OR prepregnancy OR pre pregnancy OR preconception* OR pre conception* OR periconception* OR peri conception* OR ((preterm OR premature) and (labor OR labour)) OR eclamp* OR preeclamp* OR pre eclamp* OR amniocentes* OR chorion* vill* OR breastfe* OR breast fe* OR lactation* OR cesarean OR caesarean OR cesarian OR caesarian OR cesarien OR caesarien OR newborn* OR new born* OR tocoly* OR fetal OR foetal OR fetus OR foetus OR miscarriage* OR pregnancy OR pregnancies OR pregnant).ti,ab,kf.)
