# Supplementary material for: Vaping in Pregnancy: A Systematic Review
Source: Nicotine Tob Res. 2021 Feb 4;23(9):1451–8. doi: 10.1093/ntr/ntab017 (PMC8372638; doi:10.1093/ntr/ntab017)
Supplement: ntab017_suppl_Supplementary_Materials [file ntab017_suppl_supplementary_materials.docx]

**Supplementary material**

Supplementary Table 1: Study descriptions

| **Author and year** | **Country and year of data collection** | **Study design** | **Participants** | **Funder** |
| --- | --- | --- | --- | --- |
| Ashford et al. 2016 | US  Not reported | Cross-sectional survey | 100 pregnant women and 94 non-pregnant women who used tobacco [note: authors include vaping products in this definition] in the past 12 months  Mean (SD) age in years for all 194 participants: 29.6 (6.7)  Among pregnant women: Current smoker: 57%; recent former smoker: 23%, never smoker: 10% [note: 10% missing to a total of 100%, not reported]  Among non-pregnant women: Current smoker: 93.6%; recent former smoker: 5.3%, never smoker: 1.1% | University of Kentucky |
| Bhandari et al. 2018 | US  2015 | Cross-sectional survey | 382 pregnant women  Age in years (n=376): 18-20: 16.8%; 21-24: 27.9%; 25-29: 30.0%; 30-34: 15.2%; 35-45: 10.1%  Gestational age (n=373): First trimester: 11.0%; Second trimester: 26.3%; Third trimester: 55.5%; Postpartum: 7.2%  Current smoker: 26.5%; Former smoker: 35.0%; Nonsmoker: 38.5% (of n=377) | Not reported |
| Bowker et al. 2018 | UK  2015-2016 | Cross-sectional telephone interviews  (Qualitative) | 15 pregnant, 15 post-partum women  Age range: 21 to 38 years  First trimester: 3; second trimester: 7; third: 5; 0-3 months post-partum: 6; 4-6 months post-partum: 9  Smoker: 16; Recent former smoker: 14 | Cancer Research UK |
| Cardenas et al. 2019 | US  2015 to 2017 | Cohort study of pregnant women | 248 pregnant women (a subgroup of whom were included in the study by Clemens and colleagues)  Age in years: 18-22: 37.9%; 23-27: 30.6%; 28 and over: 31.5%  Weeks pregnant: <20: 33.9%; 20 and over: 65.3%; Missing: 0.8%  Current smoker: 31.0% | Arkansas Department of Health;  University of Arkansas for Medical Sciences |
| Chiang et al. 2019 | US  2015 to 2016 | Secondary data analysis of a randomised controlled trial of a text-messaging intervention for smoking cessation with measures at baseline and after one month | 428 pregnant women who smoked  Mean (SD) age in years: 26.4 (5.8)  Mean (SD weeks pregnant: 18.1 (7.8) | National Institute on Drug Abuse of the National Institutes of Health;  George Washington University |
| Clemens et al. 2019 | US  2015 to 2016 | Biomarker analysis of hair samples from a cohort study of pregnant women | A subset of 76 women from the group of 248 described by Cardenas and colleagues  Current smoker: 50%; former smoker: 17.1%; never smoker: 32.9% (self-reported) | National Institutes of Health Clinical and Translational Science Award;  Arkansas Department of Health;  Arkansas Bioscience Institute;  Envoys, an advocacy group of the University of Arkansas for Medical Sciences Cancer Institute Foundation |
| England et al. 2016 | US  2013 | Focus groups  (Qualitative) | 102 women  Age range: 18 to 40  Smokers planning to become pregnant: 42%; pregnant smokers: 31%; pregnant recent former smokers: 26%. | Food and Drug Administration |
| Fallin et al. 2016 | US  Not reported | Focus groups  (Qualitative) | 8 pregnant and 4 newly postpartum women who smoked or used vaping products in the three months before or during pregnancy  No further information on participants provided | Not reported |
| Hawkins et al. 2020 | US  2015 to 2016 | Cross sectional survey data from the Pregnancy Risk Assessment Monitoring System (PRAMS) | 33,964 women from 29 states and New York City. Participants are recruiting using birth certificates to identify women who have delivered a live-born infant. | The authors stated that there were no financial disclosures for this paper |
| Johnston et al.2019 | UK-based forums and others  Not reported | An ‘infodemiological’ study analysing the content of online discussion forums | People posting on two UK based online parenting forums with over 1000 members.  10 discussion threads on vaping and breastfeeding  No information on participants reported | National Institute for Health Research School for Primary Care Research |
| Kahr et al. 2015 | US  Not reported | Focus groups  (Qualitative) | 87 pregnant women  No information on age, smoking status or pregnancy provided | National Institute of Health; DFG (German Research Foundation);  Baylor College of Medicine |
| Kapaya et al. 2019 | US  2015 | Cross-sectional survey, random sample of births | 3,277 women with a recent live-birth in Texas and Oklahoma  Smoking: In past 2 years: 18.5%; in 3 months before pregnancy: 16.4%; in last trimester: 6.1%; 2-6 months after delivery: 10.3%  No further information provided | Not reported |
| Kurti et al. 2017 | US  2013-2014 | Cross-sectional survey | 388 pregnant women  Age in years: 18-24: 35.9%; 25-34: 53.2%; 35-54: 10.9%  smoking prevalence 13.8%  Mean weeks pregnant: 20.9  Current smoker: 13.8%; Former smoker: 42.8%; Never smoker: 43.3%; | National Institute on Drug Abuse;  Food and Drug Administration;  National Cancer Institute; National Institute on General Medical Sciences Abuse;  National Institute of Child Health and Human Development;  Centers for Disease Control and Prevention |
| Kurti et al. 2018 | US  2013-2015 | Longitudinal survey | 7,841 women who responded to both waves. Of those, 332 were pregnant at wave 2. Characteristics reported are for wave 1 (when none were pregnant). For n=332 at wave 1:  Age in years: 18-24: 32.7%; 25-34: 59.7%; 35-54: 7.6%  Cigarettes: Established use: 19.2%, experimental use: 4.0%  E-cigarettes: Established use: 2.8%, experimental use: 2.4%  Hookah: Established use: 2.7%, experimental use: 4.5%  Cigar: Established use: 2.9%, experimental use: 3.4% | National Institute on Drug Abuse; Food and Drug Administration;  National Cancer Institute;  National Institute on General Medical Sciences Abuse;  National Institute of Child Health and Human Development;  Centers for Disease Control and Prevention |
| Kurti et al. 2020 | US  2013-2016 | Longitudinal survey | Participants were recruited from the PATH study, a nationally representative study of the non-institutionalised US population.  A longitudinal sample of 3,767 women aged between 18 and 49, of which none were pregnant at wave one and 197 (weighted % = 4.5%; CI 3.8% - 5.1%) were pregnant at wave 2. | The project was part funded by a Tobacco Centers of Regulatory Science (TCORS) award from the National Institute of Drug Abuse (NIDA) and Food and Drug Administration (FDA). It was also funded by an Institutional Training Grant award from NIDA, a Centers of Biomedical Research Excellence award from the National Institute on general Medical Sciences and Research awards from the Centers for Disease Control and Prevention (CDC) and from the National Institute for Child Health and Human Development (NICHD) |
| Liu et al. 2019 | US  2014-2017 | Cross-sectional survey | 27,920 women, of whom 1,071 were pregnant  Age range: 18 to 44 years (among all 27,920 women)  Current smoker: pregnant women: 8.0%; non-pregnant women: 14.3% | Not reported |
| Mann and Faflik 2018 | UK  2016 | Cross-sectional surveys | 36 smoking cessation services  29 pregnant women attending a smoking cessation service  Mean (SD) age in years: 28.3 (5.7)  Mean (SD) weeks pregnant: 18.4 (7.2)  Smoker: 15; Recent former smoker: 14 | Unfunded exploratory pilot work |
| Mark et al. 2015 | US  2014 | Cross-sectional survey | 316 pregnant women,  Mean (SD) age in years: 25.6 (5.5)  Mean (SD weeks pregnant: 28.3 (8.3)  Current smoker: 15.0%; Ever smoker: 43% | Not reported |
| McDonnell et al. 2020 | Ireland  2017 to 2018 | Prospective cohort study | Pregnant women in a large urban maternity hospital. 218 women who used vaping products and who did not smoke; 195 women who used vaping products and who smoked (dual users); 99 women who smoked and 108 women who neither smoked nor vaped. | The study was funded by ‘Friends of the Coombe’ research charity and by Coombe Women and Infants University Hospital. Neither funding has a role in any aspect of the study. |
| Oncken et al. 2017 | US  2012-2016 | Cross-sectional secondary data analysis of a randomised controlled trial of NRT for smoking cessation in pregnancy | 103 pregnant women who smoked  Mean (SD) age in years: 27.7 (6.0)  Mean (SD weeks pregnant: 21 (1)  Ever cigarette smoker: 74%; During pregnancy: cigarettes: 50%; hookah: 11%; cigar: 11%; marijuana: 46% | National Institutes of Health; Pfizer pharmaceuticals; University of Connecticut |
| Schilling et al. 2019 | Not reported  Not reported | A ‘netnographic’ analysis of German speaking online forums discussing vaping in pregnancy | People posting on online forums discussing vaping in pregnancy, 25 threads with 1552 posts were included.  No information on participants reported | No funding reported |
| Stroud et al. 2019 | US  Not reported | Interview study (quantitative) | 100 pregnant women, 50 smoked during pregnancy, 50 did not  Mean (SD) age in years: 26 (4)  Mean (SD weeks pregnant: 28.3 (8.3) | National Institute of Drug Abuse of the National Institutes of Health;  Food and Drug Administration |
| Wigginton et al. 2017 | Not reported  2015 | Content analysis of online forums | 13 online forum discussion threads on safety of vaping  No information on participants reported | Not reported |

*Abbreviations: NRT: nicotine replacement therapy; RCT: randomised controlled trial*

Supplementary Table 2: Patterns of use, Reasons for vaping and Smoking behaviour outcomes

| **Paper ID** | **Research question / outcome** | **Quality assessment^1^** |
| --- | --- | --- |
|  | **RQ2: patterns of use** |  |
| Ashford et al. 2016 | Frequency of vaping in 100 participants:  Daily: 3%  Often: 4%  Occasionally: 8%  Not used in last 30 days: 33%  Never used: 52% | Newcastle-Ottawa Scale: 5/9 (poor quality) |
| Bowker et al. 2018 | Participants reported vaping at home or with friends and family and said that they vaped in similar environments as for smoking. Some said they avoided vaping in front of children.  There was preference for lighter and smaller vaping products, convenience was important. Most were aware of the range of flavours available; some were aware of the range of nicotine strengths available.  Frequency of vaping out of 20 current and former users (n):  Tried only: 8  Non-daily: 5  Daily: 7 | Consolidated criteria for reporting qualitative research (COREQ): 27/32 |
| Cardenas et al. 2019  Clemens et al. 2019 | Frequency of vaping in 24 past-month vapers (n):  Daily to 10 days per month: 5  3 to 9 times per month: 7  1 to 2 times per month: 12 | NOS: 6/9 (fair quality)  *NOS: 7 (fair quality)* |
| Chiang et al. 2019 | Of 36 women who at baseline had vaped in the past 7 days, 16 continued vaping at 1 month, 20 had stopped vaping. Of 392 non-vapers (but smokers) at baseline, 14 (3.6%) started vaping. | NOS: 9/9 (good quality) |
| Hawkins et al. 2020 | Among participants who used vaping products in the last trimester of pregnancy, 43.2% used them one day a week or less. 10.4% used vaping produces on 2 to 6 days per week, 16.0% used vaping products once a day and 50.5% used them more than once per day. 0.8% of participants used both vaping products and cigarettes. | NOS: 8/9 (good quality) |
| Kapaya et al. 2019 | Among those who vaped during last trimester, 38.4% used nicotine, 35.2% no nicotine, 26.4% did not know | NOS: 7/9 (good quality) |
| Kurti et al. 2017 | Among 34 current vapers, mean numbers of days vaping in the past month: 13.1 | NOS: 9/9 (good quality) |
| Mann and Faflik 2018 | Number of days vaped in the last 30 days out of 20 who had ever tried (n):  Every day: 1  Occasionally: 3  N/A: 20 | NOS: 2/9 (poor quality) |
| Mark et al., 2015 | Among 42 ever vapers, 2 (4.8%) reported current daily use | Hoy: Moderate risk (6/10)  NOS: 6/9 (poor quality) |
| Oncken et al. 2017 | Among 14 who vaped during pregnancy:  Mean (SD) length of use: 7.5 (10.3) days  Number of times vaped per day: range from 1 to 25  Used pre-filled cartridges: n= 7 | NOS: 8/9 good quality |
| Stroud et al. 2019 | Flavours used among 16 women who vaped during peripartum period (n)  Fruit: 11, Candy: 3, Mint: 2, Tobacco: 2, Spice: 1, Coffee: 1 | COREQ: 20/32 |
|  | **RQ3: reasons for vaping** |  |
|  | ***Qualitative studies*** |  |
| Bowker 2018 | Most were motivated to quit smoking  Some felt that vaping could help them quit  Dual users of vaping products and cigarettes felt that vaping helped them to reduce their smoking  Majority believed they were less harmful (to fetus)  Reduced smoke odour  Safer for second-hand exposure  Cheaper  Replicated and substituted smoking  Encouraged to quit using vaping products by family, friends or health professional  Curiosity | COREQ: 27/32 |
| England et al. 2016 | Cheaper price  No ash  No unpleasant odours  Use in smoke-free areas  Appealing flavours  Help with smoking cessation  Being able to see the vapour  Similarities to traditional cigarettes  Less harmful  Can be used around children | COREQ: 23/32 |
| Fallin et al. 2016 | Harm reduction strategy  Preferred vaping over traditional cessation products | COREQ: 23/32 |
| Johnston et al. 2019 | Some people used vaping products to prevent returning to smoking. They used them to deal with post-partum cravings for nicotine, often triggered by the demands of motherhood, mental health issues, lack of sleep, stress, loss of identity and relationship issues.  Some had quit smoking with the intention of starting again post-partum, but had, instead, used vaping products | COREQ: 15/32 |
| Kahr et al. 2015 | **Positives**  May be used as smoking cessation device  Generally, vaping products are safer, healthier (lack of second-hand smoke, vapour, fewer chemicals, indoor use) | COREQ: 31/32 |
| Schilling et al. 2019 | **Positives**  Harm reduction potential, less harmful than cigarettes.  Potential to help smoking cessation. Partly by imitating hand to mouth behaviour. Many reported using nicotine free vaping products for this purpose.  Perceptions that vaping products were cheaper than cigarettes | COREQ: 16/32 |
| Wigginton et al. 2017 | Happy mum, healthy baby: quitting (nicotine) ‘cold turkey’ is unsafe  Abstaining from nicotine “unsafe” or “unhealthy”, significant risks to women and their babies.  Harm reduction approach to avoid stress/harm of quitting while reducing nicotine (NRT, vaping, or reduced smoking)  Vaping is the lesser of two evils  Vaping as safer than smoking  “Safe vaping” as vaping no nicotine or flavourings, researching products  Similarity of vaping to NRT | COREQ: 12/32 |
|  | ***Survey studies*** |  |
| Ashford et al. 2016 | \|  \| **Current vapers (n=49), %** \| **Former vapers (n=77), %** \| \| --- \| --- \| --- \| \| Wanted to quit smoking \| 51 \| 55 \| \| Less harmful to others \| 37 \| 13 \| \| Thought it would be less expensive \| 35 \| 26 \| \| Can use where smoking not allowed \| 35 \| 17 \| \| Less harmful to myself \| 35 \| 14 \| \| Became pregnant \| 25 \| 3 \| \| Like taste \| 23 \| 5 \| \| Saw ads promoting vaping \| 16 \| 12 \| \| Maintain / lose weight \| 2 \| 1 \| \|  \|  \|  \| | NOS: 5/9 (poor quality) |
| Bhandari et al. 2018 | Ever vaped because trying to quit smoking:  Current vapers: 70.5%  Former vapers: 54.9% | NOS: 5/9 (poor quality) |
| Chiang et al. 2019 | Among n=36 who had vaped in past 7 days:  Help me quit: 81%  Safer for me than regular cigarettes: 42%  Taste good and does not smell: 39%  Safer for my baby than regular cigarettes: 36%  Cost: 14%  Friends and family use them: 6% | NOS: 9/9 (good quality) |
| Kapaya et al. 2019 | Among n=285 who vaped around time of pregnancy  Curiosity about products: 54.0%  Help with quitting or reducing smoking: 45.2%  Less harmful to mother: 45.2%  Availability of flavours: 42.3%  Ability to get devices without nicotine: 41.4% | NOS: 7/9 (good quality) |
| Mann and Faflik 2018 | May help me quit smoking: 7/10  May help reduce cravings or withdrawal symptoms: 7/10  May help me cut down smoking: 3/10  Can use in addition to smoking: 3/10  Can use in public place where smoking not allowed: 2/10  Doesn't contain harmful chemical: 1/10  Would replace cigarettes: 1/10 | NOS: 2/9 (poor quality) |
| Mark et al. 2015 | Among 100 ever vapers:  Less harmful to users' health: 74%  Assistance with smoking cessation: 73%  Can be used in places where cigarettes banned: 55%  Taste better: 54% | NOS: 6/9 (poor quality) |
| Oncken et al. 2017 | Among 14 who vaped during pregnancy:  To quit smoking: 57%  To reduce smoking: 36%  Curiosity: 36%  Availability: 21%  Health benefits: 7% | NOS: 8/9 (good quality) |
|  | **RQ4: smoking behaviour outcomes** |  |
| Chiang et al., 2019 | 7-day point prevalence smoking abstinence at 1-month follow-up for participants who   - did not vape across both time points: 26.4% (99/375) - vaped at baseline (defined as vaping in past seven days) but not at follow-up: 25.0% (5/20) - vaped across both time points: 12.5% (2/16) - did not vape at baseline but at follow-up: 7.14% (1/14)   All participants who vaped at baseline: 19.4% (7) 7-day point prevalence smoking abstinence, did not vape at baseline: 25.8% (101), unadjusted odds ratio (95% confidence interval): 0.70 (0.30 to 1.64); adjusted: 0.79 (0.33 to 1.92).  Made quit attempt of at least one day:  Vaped at baseline: 69.4% (25); did not vape at baseline: 67.6% (265); OR (95% CI): 1.09 (0.52 to 2.28); adjusted OR (95% CI): 1.20 (0.56 to 2.55).  Reduction in cigarettes per day, mean (standard deviation):  Vaped at baseline: 3.39 (5.5), Did not vape at baseline: 3.4 (5.0). | NOS: 9/9 (good quality) |
| Johnston et al. 2019 | Some mothers had quit smoking during pregnancy with the intention of resuming post-partum. Some had then used vaping to stay smoke-free following the birth | Consolidated criteria for reporting qualitative research (COREQ): 16/32 |
| Kurti et al., 2018 | Proportion of those using the relevant product at wave 1 who reported no current use at wave 2:   \|  \| No current use at wave 2, i.e. ‘quit’, Prevalence (95% CI) \| \| \| \| \| \| --- \| --- \| --- \| --- \| --- \| --- \| \| Wave 1 use \| Cigarettes \| E-cigarettes \| Hookah \| Cigar \| Any product \| \| Overall \| 53.4 (43.2, 63.7) \| 81.3 (59.8, 100.0) \| 98.3 (94.9, 100.0) \| 88.0 (76.4, 99.7) \| 58.7 (49.2, 68.2) \| \| Established users \| 48.6 (37.5, 59.7) \| 71.2 (38.7, 100.0) \| 100.0 (100.0, 100.0) \| 83.0 (65.6, 100.0) \| 51.2 (41.3, 61.1) \| \| Experimental users \| 77.4 (56.4, 98.5) \| 100.0 (100.0, 100.0) \| 97.3 (91.9, 100.0) \| 92.4 (76.8, 100.0) \| 87.2 (74.1, 100.0) \| \|  \|  \|  \|  \|  \|  \| | NOS: 8/9 (good quality) |
| Kurti et al. 2020 | Pregnant women comprised 4.5% of the study population.  They comprised 2.8% of harm maintainers (those who smoked at wave 1 and at wave 2); 1.3% of harm reducers (those who switched from smoking to vaping between waves); and 14.5% of harm eliminators (those who quit smoking between waves and did not use vaping products). Regression identified pregnancy as a significant predictor of harm elimination but not for harm reduction. More women who became pregnant between waves (n=197) either quit smoking completely or carried on smoking than moved to vaping products. | NOS: 6/10 (fair quality) |
| Mark et al., 2015 | Mean (SD) seriousness of trying to quit smoking (range 1-10):  Ever vapers: 6.0 (4.4), never users: 5.4 (4.6), p=0.004  Ever tried to quit smoking:  Ever vapers: 92%, never vapers: 87%, p=0.54,  Mean (SD) number of quit smoking attempts:  Ever vapers: 2.8 (3.2), never vapers: 0.9 (2.9), p=0.78 | Hoy: Moderate risk (6/10)  NOS: 6/10 (poor quality) |
| Oncken et al., 2017 | Mean (SD) motivation to quit smoking (range 0-10):  vapers (n=14: 8.1 (1.3), non-vapers: 8.4 (1.8), p=0.36  Smoking quit attempts:  < 2: vapers: 29%, non-vapers: 55%  3: vapers: 14%, non-vapers: 21%  4: vapers: 36%, non-vapers: 8%  >4: vapers: 21%, non-vapers: 16%  p=0.018 | NOS: 8/10 (good quality) |

*^1^Assessed using the Consolidated criteria for reporting qualitative research (COREQ) ^26^ and the Newcastle Ottawa scale (NOS) ^25^*

*Abbreviations: NRT, nicotine replacement therapy*

Supplementary table 3: Health outcomes

| **Paper ID** | **Baby health outcomes** | **Maternal health outcomes** | **Quality assessment** |
| --- | --- | --- | --- |
| Cardenas et al., 2019 | **Risk of smallness for gestational age**  Unexposed, including to second-hand exposure (n=97): 11.3%, referent  Dual (vapes and cigarettes) users (n=17): 23.5%; RR (95% CI) = 2.1 (0.7-5.8)  Vapes only (n=6): 33.3%; RR (95% CI) = 2.9 (0.8-10.4)  Current smokers only (n=56): 23.1% RR (95% CI) = 2.0 (1.0-4.3)  After removing inconsistent self-report for ‘unexposed group’ and adjusting for maternal age and race/ethnicity  Unexposed, incl to second-hand exposure (n=64): 7.8%, referent  Dual (vapes and cigarettes) users (n=17): 23.5%; RR (95% CI) = 2.5 (0.7-8.8)  Vapes only (n=6): 33.3%; RR (95% CI) = 5.1 (1.2-22.2)  Current smokers only (n=56): 23.1% RR (95% CI) = 2.6 (0.9-7.2)  Vaping was defined as participants who used vaping products within the previous month. | N/A | Newcastle-Ottawa Scale(NOS): 6/10 (fair quality) |
| Clemens et al., 2019  Subsample of Cardenas et al | **Risk of smallness for gestational age**  By self-report:  Non-vapers non-smokers (n=38): 7.9%, referent  Smokers (n=27): 25.9%, adjusted RR (95% CI) = 3.9 (1.1 to 13.6)  Dual users (n=11): 27.3%. adjusted RR (95% CI) =3.9 (0.9 to 16.2)  By hair nicotine level: < 2.77 ng/ml: 3.3%, referent  ≥ 2.77 ng/ml: 26.1%, adjusted RR (95% CI) =7.7 (1.1 to 56.0)  By self-report confirmed by hair nicotine level:  Non-vapers non-smokers (n=25): 4.0%, referent  Smokers (n=24): 29.2%, adjusted RR (95% CI) = 7.8 (1.0 to 59.0)  Dual users (n=9): 33.3%, adjusted RR (95% CI) = 3.9 (1.0 to 69.1)  Vaping was defined as participants who used vaping products within the previous month. | **From analysis of hair samples**  Nicotine-derived nitrosamine ketone (4-(methylnitrosamino)-1(3-pyridyl)-1-butanone (NNK):  Detected in 20% of hair samples from self-reported non-vapers non-smokers, 78% of self-reported dual users and 56% of self-reported smokers.  (4-(methylnitrosamino)-1-(3-pyridyl)-1-butanol (NNAL):  Detected in 50% of hair samples from self-reported non-vapers non-smokers, 67% of self-reported dual users, 49% of self-reported smokers   \|  \| **Self-reported** \| \| \| \| --- \| --- \| --- \| --- \| \|  \| **non-vapers non-smokers** \| **dual users** \| **smokers** \| \| **Nicotine,** ng/mg  Geometric mean (95% CI)  Median  Range \| 1.1 (0.6-2.0)  0.83  0.1-44.6 \| 11.0 (3.8-31.3)  9.0  0.7-125.6 \| 10.6 (6.5-17.4)  10.7  0.8-102.4 \| \| **Cotinine,** pg/mg Geometric mean (95% CI)  Median  Range \| 0.000 (0.000-0.001)  0.000  0.0001-1.713 \| 0.153 (0.004-5.316)  0.671  0.019-20.955 \| 0.065 (0.009-0.465)  0.610  0.037-6.106 \| \| **NNK,** pg/mg  Geometric mean (95% CI)  Median  Range \| 0.003 (0.001-0.011)  0.000  0.000-42.276 \| 0.213 (0.006-7.672)  6.095  0.000-105.163 \| 0.131 (0.019-0.88)  1.299  0.000-27.192 \| \| **NNAL,** pg/mg  Geometric mean (95% CI)  Median  Range \| 0.004 (0.001-0.013)  0.000  0.000-0.929 \| 0.030 (0.002-0.395)  0.135  0.000-1.863 \| 0.005 (0.001-0.025)  0.000  0.000-1.081 \| | NOS: 7/10 (fair quality) |
| McDonnell et al., 2020 | The birthweight of babies born to mothers who vaped (3,470g ± 555g) was similar to the birthweight of babies born to mothers who did not smoke or vape (3,471 ± 504g) with the difference between the two nonsignificant (p=0.97)  The birthweight of babies born to mothers who vaped was significantly greater (P<0.001) than the birth weight of babies born to mothers who smoked (3,166 ± 502g).  The mean birth centile of babies born to mothers who vaped was the equal to the mean centile of babies born to mothers who neither smoked nor vaped (47^th^ centile). Both were significantly (P<0.001) greater than for babies born to mothers who smoked (27^th^ Centile)  Significantly fewer babies born to mothers who vaped had low birthweight (11%) compared to those born to mothers who smoked (28%, P<0.001).  A regression analysis identified that age, ethnicity, parity and socio-economic status did not alter the significance of the findings between vapers, non-smokers and smokers.  Mean gestation at delivery was similar across groups, as were mean Apgar scores.  Outcomes for dual users of vaping products and cigarettes were similar to those for smokers for mean birthweight, mean birth centile, and breastfeeding at discharge.  Current vaping was defined as participants who said yes to a question about if they currently used e-cigarettes. | Mothers who vaped had significantly higher rates of breastfeeding at discharge (48.6%) than smokers (27.2%, P<0.001). Breastfeeding rates for mothers who did not smoke or vape were significantly higher than for mothers who vaped (61.1%, P=0.03). | NOS: 8/10 (good quality) |

1 *– Assessed using the Consolidated criteria for reporting qualitative research (COREQ) ^26^ and the Newcastle Ottawa scale (NOS) ^25^*
